# 

**Published:** 1836-04

**Authors:** 


### LIST OF ORIGINAL PAPERS PUBLISHED IN THE BRITISH JOURNALS DURING THE LAST QUARTER.

### THE EDINBURGH MEDICAL AND SURGICAL JOURNAL.

*No.* 126, *Jan.* 7, 1836.

1. Clinical Report (of the Edinburgh Infirmary) for the Summer Session of 1835. By James Syme, Professor of Clinical Surgery, &c.

2. Account of a Varioloid Epidemic in Watson's Hospital in the Summer of 1835, with Remarks on the Antivariolous powers of Vaccination. By J. Bell, Esq.

3. Dissection of a Blighted Ovum, with Remarks. By William Mackenzie, M.D.

4. On Dysentery, as it Epidemically prevailed at Boston in the autumn of 1831. By James Black, M.D.

5. Observations on Continued Fever, as it occurs in the Hospitals of Glasgow. By Robert Perry, M.D.

6. Notice of some Experiments on the Vital Properties of Arteries leading to inflamed parts, and on the immediate Cause of Death by Asphyxia. By W. P. Alison, M.D.

7. On Obstructions in the soft parts to the progress of Labour. By J. T. Ingleby.

---

### THE DUBLIN MEDICAL JOURNAL.

*No.* 24, *Jan.* 1836.

1. On the Infra-Orbital Cavities in Deers and Antelopes. By Arthur Jacob, M.D.

2. Cases of Malignant Diphtheritis: with Remarks. By Edward Bewley.

3. Observations on Diffuse Céllular Inflammation, with some Remarks on Contagion. By Charles Lendrick, M.D.

4. An Essay on Laceration of the Uterus and Vagina. By J. T. Ingleby.

5. A Parallel between the Flexed and Extended Postures in the Treatment of Fractures of the Lower Extremities. By John Horston, M.D.

6. On Tympanitis occurring in Fever. By R. J. Graves, M.D.

*No. 25, March.*

7. On a peculiar Affection of the Nerves of the Teeth. By R. J. Graves, M.D. King's Professor of the Institutes of Medicine.

8. Practical Observations on some of the most common Causes of tedious Labour. By Robert Little, M.D.

9. Researches on Laennec's Vesicular Emphysema, with Observations on Paralysis of the Intercostal Muscles and Diaphragm, considered as a new Source of Diagnosis. By William Stokes, M.D. M.R.S.A. &c.

10. Observations on the Effects of Cold on the Human Body, and on a Mode of measuring Refrigeration. By Jonathan Osborne, M.D.

11. On Fistula Lachrymalis, in which a new Method of Operating is proposed; and on Injuries of the Eye from Lime and Foreign Bodies. By Arthur Jacob, M.D. M.R.S.A.

12. Observations on Trismus Nascentium. By Robert Collins, M.D.

13. Account of a Case in which Death ensued in consequence of Nitric Acid having been poured into the Ear, with a second Case in which the Epiglottis was divided in an attempt to commit Suicide. By J. Morrisson, M.D.

14. Pathological Observations on the Brain and Nervous System. By Robert Law, M.B.

15. On Puerperal Fever. In a Letter to the Editors. By Samuel Caswell, M.D.

---

### THE LANCET.

*No. 642, Dec. 19, 1835.*

1. Cases of Diseases of the Heart, elucidating certain difficulties of Diagnosis. By John Fosbooke, M.D.

2. On the Treatment of Pulmonary Consumption by the inunction of lard. By E. A. Spilsbury, Esq.

*No. 643, Dec. 26.*

3. Case of Fracture of the Fibia, with Remarks on a Leg-trough. By H. C. Sherwin, Esq.

4. Cases of Bronchial Irritation mistaken for Croup; with Remarks on the external application of Croton Oil in Cynanche Trachealis, and on the comparative freedom of Children at the Breast from Epidemics. By James Inglis, M.D.

5. On the Injection of the Nitrate of Silver for the cure of Vaginal Discharges. By George Jewell, M.D.

6. Remarks on the Report of the Dublin Committee on the Sounds and Motions of the Heart. By E. L. Bryan, Esq.

*No. 644, Jan. 2, 1836.*

7. Case of presumed Mechanical Obstruction to the Passage of Blood in the Arteries, with Remarks. By E. Crisp, Esq.

8. Case of Coxalgia producing fatal Peritonitis, with Remarks. By William Tagert, Esq.

9. On Mineral Magnetism. By R. Davidson, Esq.

*No. 645, Jan. 9.*

10. Case of Obstruction of the Rectum, with operation to form a new Anus. By J. Lindsay, Esq. Surgeon.

11. Case of Colica Spasmodica followed by Inflammation. By M. Gibson, Esq. Surgeon.

12. Remarks on Cancer of the Uterus. By S. Young, Esq. Surgeon.

13. Analysis of Diabetic Urine to discover its free Gases. By W. R. Clanny, M.D.

14. Medicinal Preparation of Manganese, and its employment in Epistaxis. By H. Osborne, Chemist.

15. Case of Amaurosis following the suppression of Feelings of Passion. By J. I. Stein, Esq. Surgeon.

*No. 646, Jan. 16.*

16. Observations on Diagnosis in Disease of the Heart.—On Palpitation, the Nervous, the Plethoric, and the Symptomatic kinds. By John Fosbroke, M.D.

17. Case and Arguments adduced to prove that the Affection called Gonorrhœal Rheumatism is produced by the use of Copaiba. By F. Eagle, Esq. Surgeon.

*No. 647, Jan. 23.*

18. On the Diminution in the Mortality of Infants in England; with a New View of the London Bills of Mortality for one hundred years, indicating a regularly-progressing Diminution. By T. R. Edmonds, Esq. B.A.

19. Cases of Vomiting cured with Creosote. By George Bodington, Esq. Surgeon.

20. Case of Fungus Medullaris in the Cavity of the Pelvis. By Julius Wolff, M.D.

21. Case of Modified Epilepsy, exhibiting a singular anomaly of the Pulse. By John Furlonge, M.D.

22. On Resuscitation from Drowning. By George Wooley, Esq. Surgeon.

23. Case of Fatal Strangulated Inguinal Hernia. By W. G. Gowing, Esq. Surgeon.

24. On Copaibal Rheumatism. By W. B. Maddock, Esq. Surgeon.

*No.* 649, *Feb.* 6.

25. Case of Small-Pox complicated with Hysteria. With Remarks on Epidemic Small-Pox at Lisburn. By John J. Kelso, M.D.

26. On Attacks of Rheumatism following Gonorrhœa. By J. B. Samuel.

27. On the Principle of Remuneration in Contracts for the Supply of Medical Aid to the Sick-Poor. By W. Fergusson, M.D.

28. Letter from Mr. Rumsey, of Chesham, respecting Measures for Altering the Poor-Law Amendment Act.

*No.* 651, *Feb.* 20.

29. Case of Gouty or Sodaic Concretions deposited in the Hands, Feet, Knee, Elbow, Nose, Cheeks, and Gums. By T. H. Barker, Esq.

30. Detection of Petroleum in the Blood. By Mr. Osborne, Chemist.

31. Remarks on the Use and Abuse of Brown Bread. By C. Wright, Esq.

32. On the Hunterian Theory of the Contamination, Disposition, and Action of the Poison of Syphilis. By F. Eagle, Esq. Surgeon.

33. Report from the Small-Pox Hospital. By George Gregory, M.D.

*No.* 652, *Feb.* 27.

34. Case of Aneurysmal Tumour in the Orbit, following Injury of the Head, and treated by Ligature of the Common Carotid. By George Busk, Esq. Surgeon.

35. Case of Severe Hemorrhage from the Mouth, of Hereditary Origin. By Julius Wolff, M.D.

36. Remarks on Mr. Osborn's Analysis of the Blood.

37. Remarks "On Dr. William's Reclamation relative to Dr. Hope's late Exposition of the Sounds of the Heart." By E. L. Bryan, Esq.

*No.* 653, *March* 5.

38. Case of Aphonia successfully treated by perseverance with Tonics and Blistering. By James Inglis, M.D.

*No.* 654, *March* 12.

39. On the Existence of Nervous Induction in functional Processes. By W. F. Bow, M.D.

40. On the Absurdities and the Merits of the Doctrines of Hahnemann. By Charles Schmidt, M.D.

41. On Ligatures in Hemorrhage. By John M. Neligan.

---

### THE LONDON MEDICAL GAZETTE.

*No.* 420, *Dec.* 19, 1835.

1. On the Defects and Results of English Parish Registers; and the comparative influence of domestic manufacture or factory labour on Female Mortality. By John Rickman, Esq.

2. On the Sources of the Vital Powers, and the relations they bear to each other. By A. P. W. Philip, M.D.

3. Question whether Vitality is either imparted or sustained by Galvanism. By J. W. Earle, Esq.

4. Observations on Arterial power in Health and Disease, particularly in Inflammation. By J. Slade, M.D.

*No.* 421, *Dec.* 26.

5. On the Functions of Taste and common Sensation in the Tongue. By Daniel Noble, Esq.

*No.* 422, *Jan.* 2, 1836.

6. Observations on the Venereal Practice of Berlin. By Alexander Ure, M.D. M.R.C.S.

7. On Electricity and Dr. Philip's Views of the Nervous Influence. By C. J. B. Williams, M.D. F.R.S. &c.

8. Miscellaneous Practical Cases. By P. M. Lyons, M.B. A.M.

Case of Malposition of the Heart, &c. with Imperforate Vagina.

Case of excessive Quantity of Opium being taken without fatal Results.

Case of General Dropsy, complicated with Enteritis.

Case of Sudden Death nineteen days after Uterine Hæmorrhage.

Case of Galactorrhœa.

9. Report of Fractures treated in the London Hospital during November. By John Adams, Esq.

10. Defence of Dr. Elliotson's employment of Iodine, and other Remedies. By W. Munk, Esq.

11. Does Iodine cause Salivation? By Jas. H. Horne, Esq.

*No.* 423, *Jan.* 9.

12. Case of Anomalous Condition of the Large Intestine and other Viscera, in a Child. By Dr. Theophilus Thompson.

13. On the Causes of Prolapse of the Funis in Labour. By John Roberton, Esq.

14. On the "Bruit de Soufflet," or Bellows Sound, in Auscultation. By Dr. Chas. Cowan.

15. Efficacy of the Chloride of Zinc in the Treatment of Malignant Ulcer. By Alex. Ure, Esq.

16. Electricity and the Nervous Influence.—A final Reply to Dr. Williams. By Dr. Wilson Philip, F.R.S.

*No.* 425, *Jan.* 23.

17. Death of an Infant from the Inhalation of Hot Turf Ashes. By G. Bury, Esq.

18. On the Average Duration and the Probability of Life, as ascertained in the several Counties of England. By John Rickman, Esq.

Tabular View of the Coincidence of the *Vie moyenne* and *Vie probable*, in relation to increase of Population.

Remarks on certain Belgian, Swiss, and Genevese Returns.

19. Second Report of Cases treated in St. George's Hospital, (being from July 1, 1835, to Jan. 1, 1836,) with "Rough Leaves" from his Case-Book. By R. Macleod, M.D.

Case in which the Semilunar Valves were perforated.

Additional Note regarding Kreosote.

Bleeding in Acute Rheumatism.

Cases of Acute Rheumatism.

Hydryodate of Potass in certain forms of Chronic Rheumatism.

Note on the rate of Mortality in St. George's Hospital.

20. Case of Fatal Hæmorrhage from Lancing the Gums. By R. T. Taynton, Esq.

21. Fatal Constipation caused by a knot of Teniæ. By J. Parkinson, Esq.

*No.* 426, *Jan.* 30.

22. Remarks on Partial Amputations of the Foot and Hand; with Cases. By John Macfarlane, M.D. Senior Surgeon to the Glasgow Royal Infirmary, &c.

*No.* 427, *Feb.* 6.

23. Observations on the Venereal Practice of Berlin. By Alexander Ure, M.D. M.R.C.S.

24. Ophthalmic Surgery.—Treatment of Eversion of the Eyelids, and of Hare's Eye. By William Brown, M.D. Lecturer on Operative Surgery of the Eye, Glasgow.

25. Some Remarks on the Modification of the Senses. By John Bishop, Esq.

26. Letter on the Blind. By Monsieur A. Rodenbeck, Member of the Chamber of Deputies, Brussels.

27. On the Causes and Treatment of Prolapse of the Funis in Labour. By R. T. Hunt, one of the Surgeons to the Manchester Lying-in Hospital,

28. Statistics and Mortality of Hospitals. By William Farr, Esq.

29. A concise Summary of the Facts relating to the Nature of the Nervous Influence. By A. P. W. Philip, M.D. F.R.S. &c.

30. Anomalous Case of Lithotomy. By Professor Lizars.

*No.* 428, *Feb.* 13.

31. Letter on the Blind. By Monsieur A. Rodenbach, Member of the Chamber of Deputies, Brussels.

32. An Account of some of the most celebrated Medical Institutions in Germany. Communicated, in a Letter from Munich, by Edwin Lee, Esq.

33. On the Use of Veratrine in the Treatment of Chronic Glandular Enlargements. By Dr. A. Turnbull.

34. An Account of Two Cases in which the Parasite of Human Muscles — the *Trichina Spiralis*—was lately found. By T. Blizard Curling, Esq.

*No.* 429, *Feb.* 420.

35. Observations on Urinary Deposits. By R. H. Brett, Esq. M.R.C.S.

36. On the Complicated Forms of Venereal Diseases. By D. H. Walne, Esq.

37. Observations on the Venereal Practice of Berlin. By Alexander Ure, M.D. M.R.C.S.

38. On the Pathology of Ramollissement of the Brain. By John Gay, Esq. M.R.C.S.

39. On the Doses of Medicine; with Remarks on the Homœopathic Method of Practice. By Dr. Leonard Stewart.

40. On the Treatment of Hydrocele by Seton. By Dr. Holbrook.

41. Remarkable Case of Hydrophobia; with an Account of the Morbid Appearances on Dissection. By Frederick Pritchard, Esq.

42. Vaccination during Lactation contraindicated; with illustrative Cases. By John Grantham, Esq.

43. On the Use of the Terms Common, Modified, and Specific Sensation; particularly with reference to the Sense of Taste. By Daniel Noble, Esq.

*No.* 430, *Feb.* 27.

44. Observations on Urinary Deposits. By R. H. Brett, Esq.

45. Case of Aneurismal Tumour in the Orbit, for which the Common Carotid Artery was tied. By George Busk, Esq. Surgeon.

46. Remarkable Case of Rupture of the Uterus, followed by complete Recovery. By John Stuart Currie, Esq.

47. Observations on the Deaths of some Eminent Philosophers of Modern Times. Delivered at the Royal College of Physicians, by Sir H. Halford, Bart.

*No.* 431, *March* 5.

48. Illustrations of the Physical Diagnosis of Diseases of the Valves of the Heart. By C. J. B. Williams, M.D. F.R.S.

49. Observations on Urinary Deposits. By R. H. Brett, Esq. M.R.C.S.

50. Case in which Death ensued in consequence of Nitric Acid poured into the Ear; also a Case in which the Epiglottis was divided, in an Attempt at Suicide. By J. Morrisson, M.D.

LIBRARY OF THE
Orleans Parish Medical Society

51. Sugar obtained from the Blood of a Patient, in Diabetes. By Charles Maitland, Esq.

*No.* 654, *March* 12.

52. Observations on Urinary Deposits. By R. H. Brett, Esq. M.R.C.S.

53. On secondary Uterine Hæmorrhage. By John Robertson, Surgeon.

54. Statistics of the Marylebone Infirmary for 1835; with Observations. By John Clendinning, A.M. M.D.

55. Case of Injury of the Spine. By Alexander Shaw, Esq.

56. On the Pathology of Hydrocephalus; with Cases. By Daniel Noble, Esq.

57. Case of fatal Hæmatemesis. By Robert H. Temple, Esq.

58. Plan of rendering the Registration of Deaths efficient for medical and statistical Purposes. By Henry Belinaye, Esq.

59. Tic Douloureux a *symptomatic* Disease. By R. M. Kerrison, Esq.

### THE LONDON MEDICAL AND SURGICAL JOURNAL.

*No.* 203, *Dec.* 19, 1835.

1. Remarks on Erysipelas. By John Langley, Esq.

*No.* 205, *Jan.* 2, 1836.

2. An Explanation of the Action of Tartarized Antimony, on Physiological Principles. By J. W. Moses, M.R.C.S.

*No.* 213, *Feb.* 27.

3. Case of Sudden Death, with Appearance on Dissection. By Henry Hargreaves, M.R.C.S.

*No.* 214, *March* 5.

4. Practical Remarks on Rupture of the Uterus. By Dr. Blicke.

5. Case of Purpura Hæmorrhagica. By H. Hargreaves, M.R.C.S.

*No.* 215, *March* 12.

6. Is Medicine an Empirical or Theoretical Science? and how far ought Pathology to regulate Practice? By Dr. Uwins.

### THE LONDON AND EDINBURGH PHILOSOPHICAL MAGAZINE.

*No.* 43, *Jan.* 1836.

1. Extracts from a Prize Essay on Iodine. By James Inglis, M.D.

2. Description of a Thermometer for determining minute differences of Temperature. By Marshall Hall, M.D. F.R.S.

*No.* 46, *March.*

3. On the Anatomical and Optical Structure of the Crystalline Lenses of Animals. By Sir D. Brewster.

### EDINBURGH NEW PHILOSOPHICAL JOURNAL.

*No.* 39, *Jan.* 1836.

1. Report respecting the Statistical Researches of Dr. Civiale on Calculous Affections made to the Académie des Sciences, Oct. 5, 1835.

### TRANSACTIONS OF THE ZOOLOGICAL SOCIETY.

*Vol.* 1, *Part* 4.

1. Description of a Microscopic Entozoon infesting the Muscles of the Human Body. By Richard Owen, Esq. F.R.S.

### LIST OF BOOKS RECEIVED FOR REVIEW.

1. New and complete Manual of Auscultation and Percussion, applied to the Diagnosis of Diseases. By M. A. Raciborski, M.D. Translated by W. Fitzherbert, B.A. London, 1835. 12mo. 5s.

2. A Manual of Aphorisms in Chemistry and Toxicology. By R. Venables, A.M. M.B. Oxon. London, 1834. 12mo. 7s.

3. A Practical Treatise on Midwifery, containing the result of 16,654 births, occurring in the Dublin Lying-in Hospital during a period of seven years. By R. Collins, M.D. late Master of the Institution. London, 1835. 8vo. 12s. 6d.

4. On Perforation and Division of permanent Stricture of the Urethra by the Lancetted Stiletes; with Observations on the Nature and Treatment of Spasmodic and Inflammatory Stricture and on various Urethral Affections. By R. A. Stafford, Surgeon to the St. Marylebone Infirmary. 3d edition, 8vo. London, 1836. 9s.

5. Illustrations of the Comparative Anatomy of the Nervous System. By Joseph Swan. Part I. 4to. pp. 27, seven plates, 10s. 6d.

6. Illustrations of the Botany and other Branches of the Natural History of the Himalayan Mountains and of the Flora of Cashmere. By J. Forbes Royle, Esq.

F.R.S. &c. Part VIII. folio, 10 plates. London, 1835. 20s.

7. A Treatise on the Diseases of the Eye and its appendages. By Richard Middlemore, M.R.C.S. Surgeon to the Birmingham Eye Infirmary, &c. 2 vols. 8vo. pp. 800-840. London, 1835. 1l. 15s.

8. Remarks on the Unity of the Body as illustrated by some of the more striking phenomena of Sympathy, both Mental and Corporeal, with a view of enlarging the grounds and improving the application of the Constitutional Treatment of Local Diseases. By George Macilwain, Member of the Royal College of Surgeons, &c. 1836, pp. 294, 8vo. 6s.

9. Rudiments of Physiology. By John Fletcher, M.D. Part II. *a.* and *b.* 8vo. pp. 130-146. Edin. 1836.

10. A Practical Treatise on Urethritis and Syphilis, including Observations on the power of the Menstruous Fluid and of the Discharge from Leucorrhœa and Sores, to produce Urethritis: with a variety of Examples, Experiments, Remedies, and Cures. A new Nosological Classification of the Venous Venereal Eruptions; each order illustrated by coloured plates, &c. By W. H. Judd, M.R.C.S. &c. 8vo. pp. 568, with 24 plates. London, 1836.

11. Guy's Hospital Reports. No. I. Jan. 1836. Edited by G. H. Barlow, M.A. Trin. Coll. Cam., and J. P. Babington, M.A. Trin. Coll. Cam. 8vo. pp. 188. 4 sh.

12. A Brief Memoir of Sir William Blizard, Knt. F.R.S. L. and E. &c. By William Cooke, M.R.C.S. 8vo. pp. 67. London, 1836. 3s. 6d.

13. St. Thomas's Hospital Reports. By J. F. Smith. No. II.

14. On Insanity; its Nature, Causes, and Cure. By W. B. Neville, Esq. London, 1836, 8vo. 10s.

15. Outlines of Human Pathology. By Herbert Mayo, F.R.S. Part I. London, 1835. 8vo. 8s.

16. First Annual Report of the Poor Law Commissioners for England and Wales. London, 1835. 8vo.

17. Illustrations of the Comparative Anatomy of the Nervous System. By Joseph Swan. Part I. London, 1835. 4to. pp. 27, 7 plates, 10s. 6d.

18. New Treatment of Malignant Diseases, and Cancer, without incision. By A. M. Riofrey, M.D. London, 1836. 8vo. pp. 78, 3s.

19. A Manual of Medical Jurisprudence and State Medicine, compiled from the latest Legal and Medical works, &c. &c. By Michael Ryan, M.D. 2d edition, considerably enlarged and improved. Lond. 1836, 8vo. pp. 554, 13s.

20. An Essay on the Laryngismus Stridulus, or Croup-like Inspiration of Infants. To which are added Illustrations of the General Principles of the Pathology of the Nerves, and of the Functions and Diseases of the Par Vagum and its principal branches. By Hugh Ley, M.D. Illustrated with plates. London, 1836, 8vo. pp. 480, 15s.

21. Illustrations of the Elementary Forms of Disease. By Robert Carswell, M.D. Fasciculus ninth. *Hypertrophy.* Lond. 1836, folio.

21. Cyclopædia of Anatomy and Physiology. Part V. (Blood—Cavity.) March, 1836, 5s.

---

### FOREIGN.

1. Ueber den Zustand der heilkunde und über die volks-krankheiten in der Europäischen und Asiatischen Türkei. Ein Beitrag zur kultur-und sittens-geschichte, Von Fried. Willh. Oppenheim, Doctor der Med. und Chir. Hamburg, 1833. 8vo. pp. 143.

2. Mittheilungen ueber die Cholera-Epidemie zu St. Petersburg im sommer 1831. 2 vols. 8vo. St. Petersburg, 1832.

3. De Phthisi Tuberculosa. Scripsit Alexander Blastos, Doctor Med. und Chir. Berdini, 1833. 8vo. pp. 136.

4. Nouvelles Recherches sur le Rhumatisme Aigu en général, et spécialement sur la loi de coincidence de la péricardite et de l'endocardite avec cette maladie, &c. Par J. Bouillaud, Professeur de Clinique, &c. Paris, 1836. 8vo. pp. 162.

5. Medicinisch-praktische Abhandlungen, von Deutschen in Russland lebenden Aertzen. Ersten Band. Hamburg, 1836. 8vo. pp. 432.

6. Untersuchungen zur Physiologie und Pathologie. Von Dr. F. Nasse. Heft I. II. Bonn. 1835. 8vo. pp. 316.

7. Einige Bemerkungen über den einfluss der witterrung auf den menschlichen organismus uberhaupt. Von Dr. B. Becker. Parchin, 1835. 8vo. pp. 89.

8. De Suicidio, Observationibus Anatomico-Pathologicis Illustrato. Scripsit D. J. A. Arntzenius, Medicinæ et Chirurgiæ Doctor. Trajecti ad Rhenum, 1835. 8vo. pp. 204.

9. Disquisitio Physiologica de differentia et nexu inter nervos vitæ animalis et vitæ organicæ. Auctore J. Van Deen, M.D. Lugduni-Batavorum, 1834. 8vo. pp. 183.

10. Ueber Volkskrankheiten und deren Bekämpfung. Von. E. L. H. Lebenheim, Doctor Med. et Chir. Hamburg, 1836, 8vo. pp. 144.

---

### INDEX TO VOL. I.

OF THE

### BRITISH AND FOREIGN MEDICAL REVIEW.

PAGE

A.

ABDOMINAL PLETHORA, a remote cause of phthisis . . 77
Abdomen, bibliography of . 515
Absorption, Dr. Jackson on . 156
Aconite, its effects on animals 249
Adipose tissue, Dr. Geddings on . 153
Age, its effects on the structure of the lungs . . . 233
Air in the veins, death from, in operations . . . 162
Almanac, British medical, notice of 216
Aldis, Dr. his introduction to hospital practice . . . 490
Alum, its use in typhoid fevers 568
Alibert on the treatment of porrigo 578
American Medical Journals . 228
Cyclopædia of Medicine and Surgery . . . 152
Anatomy Morbid, Dr. Carswell's Illustrations of . . . 117
Aneurism of the aorta, treatment of 161
Andral, translation of his Clinique Medical . . . 188
Anatomy of the Heart, Bouillaud on the 420
and Physiology, Cyclopædia of 536
of the Head and Neck, Mr. Cock on . . . 541
Aorta, diseases of, Dr. Geddings on 157
Armstrong, Dr. his life, by Dr. Boot 34
his lectures, by Mr. Rix ib.
his rejection by the College of Physicians 54
his death and character 66
Areola of the nipple as a sign of pregnancy . . . 100
Arnold, on the structure of bone and cartilage . . . 231
Arnold, on the origin of the cervical nerves . . . 550
Arnold, on the development of the iris 551
Arteries, abnormal sounds of 456-7
Asylum, Lunatic, on an improved plan 308
Association, British, reports of . 368
Atmosphere, its influence on health 345
Auscultation in pregnancy, its uses 87-95
Dr. Raciborski on . 479
Bibliography of . 515

PAGE

B.

Ballota lanata, its use in gout and rheumatism . . . 566
Baer, Prof. on the development of the embryo . . . . 238
Balmanno, Dr. dinner in Glasgow to 307
Bardsley, Dr. his Introductory Address 206
Barry, Sir D. biographical notice of 611
Baths and bathing, in relation to hygiène . . . . 336
bibliography of 516
Baring, Dr. Otto, on fungous testicle 469
Bene, Dr. his Elementa Medicinæ . 1
Becquerel and Breschet, on the temperature of the body in disease . 246
Belgium, Medical Association of . 607
Beds and bedrooms, in relation to hygiène . . . . 335
Bellows sound, or *bruit de soufflet*, its nature . . . . 451
Benzoin, fumigations of, in hooping cough . . . . 574
Bibliography, Medical, Dr. Forbes's Manual of . . . 513
Bloodletting in fever, history of . 44
M. Louis on . 397, 405
Black discoloration, Dr. Carswell on 135
Blenorrhagia in women, treatment of 271
Blood, researches on, by Gmelin, &c. 590
state of, after removal of the kidneys . . . 592
Boot, Dr. his Life of Dr. Armstrong 34
his treatise on Fever . 67
Botany of the Himalayan mountains 215
Bone and cartilage, structure of . 231-549
Books received for review, list of, 310, 619
Bouillaud, M. his treatise on Diseases of the Heart . . . 424
Boehm, Dr. on the anatomy of the intestinal glands . . . 521
Bones, on the structure of, by Medici 549
Brain, chemical analysis of . 288
and nerves, experiments on, by Mayer . . . . 557
Bronchocele, bibliography of . 517
Brunner's glands, anatomy of . 527
Brera, Prof. on the use of the ballota lanata . . . . 566

 T T

PAGE
Broomseed, its use in dropsy . 533
Buckingham, Mr. his Bill for public walks, &c. . . . . 304
Burns, Velpeau on the treatment of 579
Dr. Hintze on the treatment of 581

C.

Cachexy, tuberculous, Dr. Clark on, 72, 74
Catamenia, age of appearance of . 102
Carswell, Dr. his illustrations of Pathological Anatomy . . 117
Carcinoma, morbid anatomy of, by Dr. Carswell . . . 127-132
Cartilage and bone, on the structure of 231
Carbonic acid gas, solidification of . 288
in the blood . . 590
Cæcum, Unger, Ferrall, and Smith on the . . . . . 501
Cephaloma, Dr. Carswell on . 128
Cerebral hemorrhage, its relation to paralysis . . . . 142
Cephalæmatoma, Drs. Unger and Geddings on . . . . 182
Cervical nerves, origin of . . 550
Cholera, use of cold drinks in . 15
anatomical characters of . 236
infantile, use of alumina in 257
Chiappa, Dr. Del, his Clinical Report 249
Chevallier on the diseases of printers 282
Cheyne, Dr. biographical notice of . 614
Ciliary motions in animals, Purkinje and Sharpey on . . . 509
Clark, Dr. J. his treatise on Consumption . . 70
Dr. W. his report on Physiology . . . 379
Clot-Bey on the plague of Egypt . 248
Climate of Guiana, recommended in Phthisis . . . 508
Clinical instruction, M. Louis on . 400
Clothing in relation to hygiène . 332
Consumption, Dr. Clark's treatise on 70
statistics of . 78
prevention and treatment of . 81-2
Corporations, Medical, British, statistics of . . . . 306
Irish, statistics of 610
Combe, Dr. his Principles of Physiology . . . . . 328
Contagion, on the origin and laws of 369
Cooper, Sir A. on fungous testicle 471-8
Cock, Mr. his Anatomy of the Head and Neck . . . . 541
Copaiba employed as an enema in gonorrhœa . . . . 578
Cowan, Dr. his translation of Louis on Phthisis . . . 165
Cramer, Dr. on the cure of fistulæ and ulcers . . . 268
Croup, treatment of, by sulphate of copper . . . . 568
Creosote, its medicinal effects . 265

PAGE
Cutaneous diseases, Dr. Bene on . 12
Cyclopædia of Practical Medicine, notice of . . . . 176
American, of Medicine, &c. 152
of Anatomy and Physiology 536

D.

Danish Medical Journals . . 227
Deposits of pus in internal organs, of the . . . . 149
Development, organic, theory of . 389
Dentists in Prussia, qualifications of 608
Dictionaries, Medical, account of the principal . . . . 176
Diabetes, case of, cured . . 367
Diagnosis of Pregnancy, Hohl and Kilian on the . . . 85
Dictionary of medical terms, Hoblyn's 517
Dislocations, secondary treatment of 583
of the clavicle, Velpeau on, 584
of the shoulder-joint, M. Malgaigne on . 585
Dropsy, Dr. Bene on . . 20
Dürr on alumina in infantile cholera 257
Dublin Journal, original papers in the 309
Dunglison, Dr. his Elements of Hygiène . . . . 344
Ducros, M. on turpentine enemata in sciatica . . . . 569
Dysentery, use of nux vomica in . 255
Dyspepsia a remote cause of phthisis 73

E.

Edinburgh Medical Journal, original papers in . . . . 309
Dictionary . 178
Education, medical, remarks on . 2
Elementary Forms of Disease, Dr. Carswell on . . . 117
Embryo, early development of . 238
Emetic tartar as a remedy, M. Louis on . . 397-405
M. Lepelletier on, 379-415
Esquirol on the statistics of insanity 272
Everitt, Dr. on stramonium in sarcocele . . . . 578
Exploration, Obstetric, Hohl and Kilian on . . . 85
Exhalation, Pulmonary, Tiedemann on 241
Exercise, its hygienic importance . 354

F.

Fever, Dr. Bene on . . 1
Dr. M'Cormac on . . 172
Febrile and inflammatory diseases, difference of . . . 30
Ferrall, Mr. on phlegmonous tumour of the cæcum . . . 501
Fistulæ and ulcers, Dr. Cramer's treatment of . . . . 268
Fingers, permanent retraction of . 269
Flannel, its hygienic value . . 334

PAGE
Fitzherbert, Mr. his translation of Raciborski . . . 479
Fœtal heart, pulsation of, a sign of pregnancy . . . 89
Fomites, state of our knowledge respecting . . . . 377
Forbes, Dr. his Bibliography . 513
French Medical Journals . . 223
Institutions, Mr. Lee on 449
practice, account of 495
Froriep, Dr., on purulent metastasis 569
Fungoid disease, Dr. O. Baring on . 469
Fungous and scirrhous testicle, diagnosis of . . . . 474

G.
Gama, M. and the King of Sweden 305
Geddings, Dr. on the adipose tissue 153
on diseases of the aorta 157
on bloody tumours of the scalp . 182
German Medical Journals . 217-545
German Medical Institutions, Mr. Lee's account of . . 499
Geddings, Dr. on hemorrhoidal tumours 579
Glands, intestinal, minute structure of 521
Gmelin, his researches on the blood 590
Governesses, their unhappy condition 339
Gout, use of ballota lanata in . 566
Gonorrhœa, treatment of, by copaiba enemata . . . 578
Growths, Morbid, their origin . 120
Gregory, Prof. James, his Conspectus 520
Griffith, Mr. his treatise on Hydrocephalus . . . . 535
Gunther, Dr. on nature and art in curing diseases . . 191
Guiana, Dr. Hancock's observations on 508
Guy's Hospital Reports . 543

H.
Hamilton, Dr. James, his character as a teacher . . . . 37
Hays, Dr. his American Cyclopædia 152
Hall, Dr. M. his Introductory Lecture 203
Haller, his physiological opinions 386
Hancock, Dr. his observations on guiana 508
Hæmatocele, confounded with fungous testicle . . . 476
Hemorrhage, Dr. Carswell on 141
Heart, effect of different medicines on the . . . . . 264
Heron, Dr. on ozœna . . 270
Hecker, Dr. on the state of medicine in Prussia . . . 289
Health, Philosophy of, by Dr. Smith 315
Henry, Dr. on the laws of contagion 369
Heart, diseases of, M. Bouillaud on the 424
from spinal irritation 459
its weight and size . 433
Hernia of the appendix cæci, case of 583
treated by the suction pump 586
Hohl, Dr. his work on obstetric exploration . . . 85

PAOE
Hemorrhoids, an ointment for 579
Hourmann, M. on the structure of the lungs . . . 233
Horner, Dr. on the anatomical characters of cholera . . . 236
Holst, Dr. on the state of Lunatics in Norway . . . 278
Hospitals in Paris, statistics of 281
Hodgkin, Dr. on the means of promoting health . . 362
Hoblyn, Mr. his dictionary of Medical Terms . . . . 517
Hospital Reports, Guy's and St. Thomas's . . . . 543
Hourmann and Dechambre on the pulse of the old . . . 553
Hooping cough, cured by fumigations of Benzoin . . . 574
Hygiène, Public and Private, various works on . . . 313
Elements of, by Dr. Dunglison 344
and Therapeutics, Dr. Kilgour on 352
Hydrocephalus, Treatise on, by Mr. Griffith . . . 535
Hydrated oxide of iron, an antidote to arsenic . 572-3, 594-5

I.
Inflammation, Dr. Bene on . 8
proximate cause of 11
Inflammatory and febrile disease, difference of . . . 30
Intus-susception, mortification from 146
Insane, moral management of the 286
Intermittents, employment of salicine in 570
Irritation, spinal, cause of various diseases . . . 459
Iris, development of the . . 551
Italian Medical Journals . 221 546
Italian Medical Institutions, Mr. Lee on 497

J.
Jackson, Dr. on Absorption . 156
Dr. James, jun. memoirs of 209
James, Dr. his Medical Dictionary 176
Johnstone, Dr. his arrangement of the Materia Medica . . 193
Journals, Foreign Medical, account of . . . . 217-545

K.
Kilgour, his lectures on Hygiène . 352
Kilian, Dr. on Obstetrical Exploration 85
Kidneys, extirpation of, effect on the blood of . . . . 592
Kluge, Dr. on the pneumonia of infants 253
Koehler, Dr. on incarcerated hernia 586

L.
Law, Dr. his address to the Birmingham Medical School . . 206
Lancet, the, original papers in . 309
Latin, the Medical Student's Guide to 579
Leeches, historical account of, in France . . . 281

PAGE

Lee, Mr. on the medical institutions of the continent . . . 494
Lefevre, Dr. his History of Medicine in Russia . . . 597
Ligaments, Mr. M'Nab's treatise on 215
Lieberkühn, glands of, anatomy of 526
Louis, M. his researches on phthisis 165
his method of studying diseases . . . 168
on clinical instruction . 400
on bloodletting and emetic tartar . . . 405
Lombard, Dr. on the influence of professions on life . 195
on nux vomica and aconite 249
on the effect of medicines on the heart . . 264
Longevity and happiness connected . 319
Lungs, diseases of, caused by spinal irritation . . . 459

M.

Macrobin, Dr. his introduction to medicine . . . . 207
Malaria, Dr. Dunglison on . . 349
Malgaigne, M. on dislocations . 583-5
Marsden, Dr. biographical notice of . 615
Martin, Mr. P. his translation of Louis's work . . . 396
Marshall, Dr. on spinal irritation . 459
Mayer, Prof. on the brain and nerves 557
Materia Medica, Dr. Johnson's syllabus of . . 193
(Museum of, in Edinburgh) 307
Mc Cormac, Dr. on fever . . 172
Mc Nab, his Compendium of the Ligaments . . . . 215
Melanoma, Dr. Carswell on . . 135
Medical men, comparative mortality of 201
Medical Gazette, original papers in . 310
corporations . . 306-610
and Surgical Journal, original papers in . . 311
Medullary fungus, Dr. Baring on . 469
Medici, Prof. on the structure of bone 549
Metastasis, purulent, theory of . 569
Mind, over exertion of, its injurious effects . . . . 341
Milliners, their unhappy and diseased state . . . . 366
Michaelis, Dr. on reposition of the funis . . . . 588
Midwifery, Dr. Reid's Manual of 512
new Journal of . . 545
cases of, in the Maison d'Accouchement . 589
Mortification, Dr. Carswell on . 144
Motherby, Dr. his Medical Dictionary 177

N.

Nervous Diseases, Dr. Bene on . 23
and vascular systems, relations of 393
Neuralgia, use of laurocerasus in . 263

PAGE

Nervous system, a source of organic disease . . . 459
Numerical method, M. Louis on the . . . 170-403
Nux Vomica, its effects on animals . 249
use in dysentery . 255

O.

Obstetrical exploration, Dr. Hohl on 85
Obituary . . . 308-611
Oppenheim, Dr. his new Medical Journal . . . . 546
Original papers in the British journals . . . 309,615
Ozœna, treatment of . . 270

P.

Parise, M. on Rheumatism . . 254
Parr, Dr. his Medical Dictionary . 177
Palate-suture, Dr. Smith on . . 265
Pathological anatomy, its different kind . . . . 118
, Dr. Carswell's Illustrations of . . . 117
Pearson, Dr. on broomseed in dropsy . 533
Percussion, Dr. Raciborski on . 479
Pericarditis and Carditis, signs of . 10
Hemorrhagic, account of 259
Peyer, glands of, anatomy of the . 522
Phlegmonous tumours of the iliac region, account of . . . 501
Phthisis, Dr. Bene on . . 22
Dr. Clark on . . 70
Louis on . . . 165
Physiology applied to health, Dr. Combe on . . . 328
History of . . 379
Animal, Dr. Clark's report on . . . . ib.
Pinel, M. unchaining the lunatics . 286
Placental sound in pregnancy . 90
, cause of . . 485
Plague in Egypt, Clot-Bey on the . 248
Pleischl, Dr. on the use of salicine . 570
Plates, Dr. Carswell's anatomical . 152
Pneumonia in infants, its character . 253
effects of bloodletting in . 405
emetic tartar in 414
Poisoning, means of preventing . 596
by mistake . . 306
by Arsenic, cases of 570-571
Porrigo, treatment of, in Paris . 578
Pott's vertebral disease, M. Richet on, 574
Pregnancy, diagnosis of . . 107
Printers, diseases of . . . 282
Professions, influence of, on mortality, 195
Provincial medical and surgical association, transactions of . . 529
Prussia, medical institutions of . 289
Puerperal Fever, Dr. Armstrong on . 39
Pulmonary consumption, Dr. Clark on . . . . . 70
Pulsation of the heart, cause of the sound of the . . . 437

26

PAGE
Pulsation of the fœtal heart, a source of diagnosis . . . . . 98
Pulse of old persons, observations on the . . . . . 553
Purkinje, on ciliary motions in animals 509
Pus, Dr. Carswell on . . 147

R.
Raciborski, Dr. his Manual of Auscultation . . . . . 479
Reid, Dr. his Manual of Practical Midwifery . . . . 512
Respiration of old persons, observations on the . . . . 553
Rheumatism, pathology of . . 254
use of ballota lanata in . 566
Ricord, M. on the treatment of blenorrhagia . . . . 271
Rix, Mr. his edition of Dr. Armstrong's Lectures . . . . . 34
Rochoux, M. on the primary form of tubercles . . . . . 238
Royle, Mr. his Botany of the Himalaya Mountains . . . . 215
Russia, history of medicine in . 597
Rust's Magazine, notice of . . 545
Rhythm of the heart, M. Bouillaud on 448

S.
Saliva, experiments on, by Schultz . 551
Salicine, its use in intermittents . 570
Sarcocele, use of stramonium in . 578
St. Thomas's Hospital Reports 203,208,543
Schultz, Prof. his experiments on saliva 581
Scrofula, Dr. Bene on . . 19
Scirrhoma, Dr. Carswell on . . 128
Sciatica, on turpentine enemata in . 569
Seidlitz, Dr. on hemorrhagic pericarditis . . . . . 259
Sharpey, Dr. on ciliary motions in animals . . . . . 509
Siebergundi, Dr. on viper bite . 582
Sinapisms, proper temperature of . 570
Skin, importance of, in the animal economy . . . . 329
Small-pox and cow-pox, are they identical? . . . . 376
Softening, Dr. Carswell on . . 137
Sounds of the heart, theories of 440--13
Smith, Dr. on palate-suture . . 265
Dr. F. on a disease of the cæcum . . . . 501
Spain, medical reform in . . 608
Spider, anatomy of . . . 538
Spillan, Dr. his translation of Andral 188
Spinal irritation, Dr. Marshall on . 459
disease, cases of . . 463
Spleen, on the diseases of, in India . 563
PAGE
Spirit drinking, evil effects of . 364
Stafford, Mr. on permanent stricture . 534
Staphyloraphy, Dr. Smith on . 265
Stethoscope, Dr. Hohl's modification of . . . . . 87
Stomach, softening of, Dr. Carswell on 138
diseases of, from spinal irritation . . . 459
Stricture, permanent, Mr. Stafford's treatment of . . . 534
Stramonium, its use in sarcocele . 578
Syphilis, Dr. Bene on . . 17

T.
Taramelli, Dr. on hernia of the appendix cæci . . . 583
Temperature of the body in disease . 246
Tiedemann on pulmonary exhalation . 241
Tonsils, extirpation of . . 267
Touch (Le Toucher), its use in the diagnosis of pregnancy . . 104
Travers, Mr. his Introductory Lecture 209
Tubercle, pathological anatomy of . 123
Tubercles, primary form of . . 238
Tuberculous cachexia, Dr. Clark on 72
Tumours, bloody, of the scalp, in infants, on . . . 182
Twining, Mr. W. biographical notice of . . . . . 613
Typhous fever, Dr. Armstrong on . 42
Typhus, alum employed in . . 568

U.
Umbilical cord, on the reposition of . 588
Underwood, Mr. his Medical Student's Guide . . . . . 519
Unger, Dr. K. on bloody tumours of the scalp . . . . 182
Unger, Dr. L. H. on diseases of the cæcum . . . . 581

V.
Vaccination in Prussia, encouragement of . . . . . 609
Vertebral disease of Pott, M. Richet on the . . . . 574
Viper bite, case of . . . 582
Voigt, Dr. on the diseases of the spleen in India . . . 563

W.
Weights, medical, comparative view of, in different countries . . 609

Z.
Zimmermann, Dr. on sulphate of copper in croup . . . . 568

END OF VOL I.

LONDON:
PRINTED BY J. AND C. ADLARD, BARTHOLOMEW CLOSE

